# Leveraging unlabeled SEM datasets with self-supervised learning for enhanced particle segmentation

**DOI:** 10.1038/s41524-025-01802-3

**Published:** 2025-09-22

**Authors:** Luca Rettenberger, Nathan J. Szymanski, Andrea Giunto, Olympia Dartsi, Anubhav Jain, Gerbrand Ceder, Veit Hagenmeyer, Markus Reischl

**Affiliations:** 1 Institute for Automation and Applied Informatics, Karlsruhe Institute of Technology, Eggenstein-Leopoldshafen, Germany; 2 Department of Materials Science & Engineering, University of California, Berkeley, Berkeley, CA USA; 3 Division of Materials Science, Lawrence Berkeley National Laboratory, Berkeley, CA USA; 4 Energy Technologies Area, Lawrence Berkeley National Laboratory, Berkeley, CA USA

**Keywords:** Batteries, Structural properties, Synthesis and processing, Materials for energy and catalysis, Theory and computation, Computational methods, Engineering, Materials science

## Abstract

Scanning Electron Microscopes (SEMs) are widely used in experimental science laboratories, often requiring cumbersome and repetitive user analysis. Automating SEM image analysis processes is highly desirable to address this challenge. In particle sample analysis, Machine Learning (ML) has emerged as the most effective approach for particle segmentation. However, the time-intensive process of manually annotating thousands of SEM images limits the applicability of supervised learning approaches. Self-Supervised Learning (SSL) offers a promising alternative by enabling knowledge extraction from raw, unlabeled data. This study presents a framework for evaluating SSL techniques in SEM image analysis, focusing on novel methods leveraging the ConvNeXtV2 architecture for particle detection. A dataset comprising 25,000 SEM images is curated to benchmark these proposed SSL methods. The results demonstrate that ConvNeXtV2 models, with varying parameter counts, consistently outperform other techniques in particle detection across different length scales, achieving up to a 34% reduction in relative error compared to established SSL methods. Furthermore, an ablation study explores the relationship between dataset size and SSL performance, providing actionable insights for practitioners regarding model selection and resource efficiency. This research advances the integration of SSL into autonomous analysis pipelines and supports its application in accelerating materials science discovery.

## Introduction

Desktop Scanning Electron Microscopes (SEMs) and advancements in laboratory automation have greatly accelerated material characterization, generating vast quantities of high-resolution images^1,2^. While this surge in data has opened new avenues for research, it has also presented significant challenges in efficient analysis^3^. For example, identifying individual particles (segmentation) in SEM images is often critical in characterizing powder samples. Traditionally, this process has been carried out manually or through basic computational methods such as thresholding, edge detection, or the application of hand-crafted image kernels^4–7^. While these approaches have been valuable, they often fall short when dealing with complex or noisy images, necessitating more sophisticated techniques. In recent years, Machine Learning (ML) approaches, particularly Convolutional Neural Networks (CNNs), have emerged as the de facto standard for computer vision tasks^8,9^, including SEM image segmentation^10^.

Recent trends in ML emphasize the importance of large, carefully labeled datasets to achieve state-of-the-art results. The ImageNet^11^ dataset, with over 14 million annotated images, exemplifies this approach and has significantly advanced the field of ML. Models initially trained on the large set of diverse images in ImageNet show impressive results when applied to new tasks, even in areas seemingly unconnected to the original training data^12^. This transferability suggests that obtaining general task solution models for specific domains, such as SEM images, may be possible if extensive annotated training examples are available. The usual approach for this task is supervised learning, where models are trained on labeled data to make predictions. However, in specialized domains requiring expert knowledge, such as the segmentation of individual particles in SEM images, annotating datasets with thousands or even millions of samples is generally unfeasible due to the complexity and expertise required for accurate annotations^3,13^. Fortunately, new methods are emerging to reduce dependency on labeled data and bridge the gap between the large quantities of available data and the inability to annotate them.

Multiple label-efficient paradigms have been introduced to address the annotation bottleneck in domains such as SEM image segmentation. Among these, Few-Shot Learning (FSL) is used to segment data based on only a small number of labeled examples, thereby reducing the need for large annotated datasets^14^. Zero-shot strategies, enabled by recent advances in foundation models, involve transferring general-purpose segmentation models, such as the Segment Anything Model (SAM)^15^, to microscopy images and refining their outputs through domain-specific post-processing^16^. In addition, Self-Supervised Learning (SSL) has been introduced as a method for utilizing large amounts of unlabeled data, enabling the creation of robust feature representations and inference models for tasks where labeling all samples in a dataset is impractical^17^.

Within these paradigms, clear trade-offs are observed: FSL methods depend on minimal but specifically chosen annotations; zero-shot strategies exploit the broad capabilities of foundation models with little to no new annotation, yet rely on large, general-purpose pretrained models whose substantial computational requirements can hinder local deployment and may necessitate reliance on external services; and SSL methods need large volumes of unannotated datasets to derive models for domain-specific tasks algorithmically. The focus of this work is placed on SSL due to its suitability for large microscopy datasets and its capacity to extract meaningful features from complex, unlabeled data. This approach is considered advantageous, as it enhances the robustness of ML systems through task-agnostic knowledge transfer while reducing dependence on extensive annotations^17^.

Recent developments in SSL methods have been extensively explored across diverse domains, demonstrating effectiveness in leveraging unlabeled data for complex computer vision tasks. Examples include the identification of microparticles in wastewater treatment systems, where SSL has been combined with limited labeled data to enhance detection accuracy^18^, and the detection of floating litter in freshwater bodies^19^, which was shown to improve generalization under varying environmental conditions. These findings underline the broad applicability of SSL techniques and their ability to reduce dependence on large annotated datasets while maintaining robust feature learning in challenging imaging scenarios. In the field of materials science, SSL has likewise proved effective in microscopic image analysis, notably through optical microscopy applications for alloy microstructure segmentation^20^ and general frameworks for diverse microscopy datasets^21^. However, existing methods face key limitations: some depend on cross-modal data or focus narrowly on optical microscopy, hindering their adaptation to SEM-specific tasks, while others assume access to large-scale labeled/unlabeled datasets and prioritize classification over segmentation, leaving critical challenges in segmentation-specific scenarios for SEM images unresolved.

**Fig. 1:**
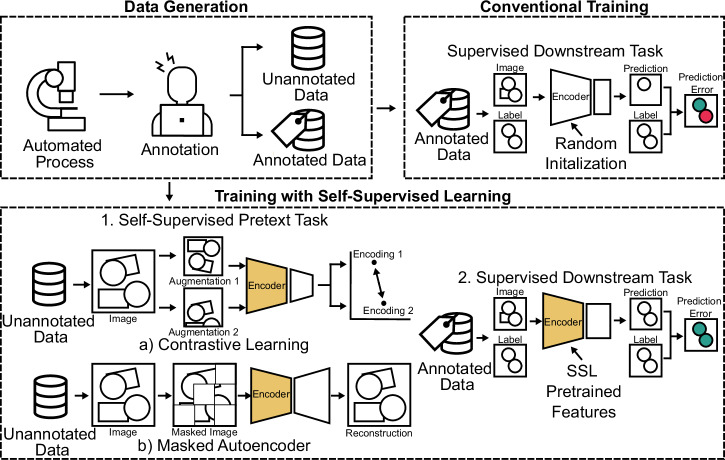
Overview of the SSL concept. An automated process generates data that is partially annotated by hand, resulting in both annotated and unannotated data. In conventional training, only annotated data is used. The encoder, responsible for learning relevant image features, is randomly initialized and used to solve the downstream task (e.g., segmentation of particles in SEM scans). This often leads to suboptimal results as sufficient data is lacking. SSL can overcome data scarcity by using labeled and unlabeled data in two steps: (1) a self-supervised pretext task where the encoder learns features from raw, unannotated data. Two approaches are shown: **a** in (CL), augmented views of the same image are encoded to be similar, and **b** in (MAEs), masked images are reconstructed. (2) The pretrained encoder is then used in the supervised downstream task with annotated data, improving performance compared to random initialization.

Figure 1 illustrates the concept of SSL. An automated process generates data, which an expert partially annotates. In conventional training, only the annotated data is used, often leading to suboptimal results when the amount of training data is insufficient. SSL utilizes both labeled and unlabeled data in a two-step process. Firstly, a self-supervised pretext task is solved, where the encoder, a neural network component that transforms input data into a compact representation, learns features from raw, unannotated data. For this, two paradigms are shown: (CL), where augmented views of the same image are encoded to be similar, and (MAEs), in which random patches of the image are hidden and reconstructed by the model. The pretrained encoder, equipped with learned features, is then used in the supervised downstream task with annotated data, significantly improving performance compared to random initialization, which is the traditional training approach.

Among the available SSL methods for computer vision, CL has gained widespread adoption and demonstrated effectiveness across a variety of tasks^22^. This technique has shown promise in leveraging unlabeled data to improve model performance even in complex domains with relatively few samples. CL methods are predominantly tailored for usage with CNNs, which are the standard neural networks for ML-based computer vision tasks. However, recent studies have revealed that (ViTs) can outperform CNNs in various computer vision tasks^23,24^, which led to the development of SSL techniques with ViTs. Using ViTs, the MAEs paradigm has seen great success, which was previously dismissed as ineffective with CNNs compared to CL^22^. However, as ViTs typically require hundreds of thousands of images to establish their potential, CNNs, and hence CL, remain the prevalent choice in SSL^25,26^. Recognizing this shortcoming of ViTs, the ConvNeXt architecture was recently introduced^27^, which modernizes CNNs by incorporating design principles from ViTs, achieving comparable performance to actual ViTs while maintaining the computational efficiency characteristic of CNNs. Building upon ConvNeXt, ConvNeXtV2 introduces architectural innovations that enable effective SSL using MAE, a technique previously ineffective with CNNs, and implements methods to enhance the quality of learned features^28^. Details regarding the ConvNeXt and ConvNeXtV2 architectures are available in “ConvNeXtV2 architecture”.

Despite the significant advancements in ML and computer vision techniques, several opportunities remain, particularly in the context of SEM image analysis and particle segmentation: (1) The exponential growth in raw data production from automated laboratories has resulted in a vast wealth of data with immense potential yet to be utilized^29,30^. (2) Recent developments in SSL techniques have shown promise in various domains but have not been thoroughly evaluated on SEM data, particularly for advanced architectures like ConvNeXtV2. (3) Generally, the evaluation of ConvNeXtV2 has been limited, focusing only on comparisons with a single CL method, Momentum Contrast (MoCo)^31^, and classification performance on ImageNet, rather than real-world data or segmentation tasks, even though it shows potential and theoretically offers numerous advantages compared to other SSL methods^28^. (4) Furthermore, the position of CNN-based MAE approaches within the SSL landscape has yet to be determined, leaving the claim that masked image modeling outperforms the prevalent CL approach in SEM unverified. (5) The current SSL research assumes that training databases contain millions of samples, which is unrealistic in many real-world scenarios, making the integration of SSL techniques into existing workflows challenging for researchers and practitioners. (6) Additionally, there is limited practical guidance and best practices on crucial aspects such as selecting optimal methods, determining appropriate model complexity, and identifying necessary dataset sizes for effective implementation, hindering SSL’s widespread adoption and utilization in SEM image analysis.

The presented study addresses shortcomings in the literature on SEM image analysis by providing solutions to efficiently handle vast amounts of raw data, thereby bridging the gap between theoretical advancements in automation and practical applications in materials characterization. The key contributions of this work are as follows. (1) Showcasing a toolbox for evaluating SSL methods in materials science, enabling qualitative and quantitative assessments of competing approaches under boundary conditions. (2) Providing practical guidance for optimizing neural network parameters, addressing computational constraints, and determining the necessary data points for model development in material science. (3) Exemplifying the evaluation approach with a comprehensive study on SEM imaging data for particle segmentation, highlighting method capabilities and limitations^3^. (4) Introducing a novel SEM dataset of nearly 25,000 images from inorganic powder, challenging the notion that millions of samples are required for effective SSL. (5) Evaluating the performance of MAEs versus CL pretrained on the novel dataset and ImageNet, providing insights into their relative strengths in SEM image analysis and advancing the understanding of SSL techniques in the field while also assessing whether a specialized, albeit smaller, SEM dataset can compete with extensive databases. (6) Exploring optimal configurations for SSL with SEM data by assessing model sizes, trainable parameters, and computational constraints. (7) Deriving generalizable insights into neural networks’ learned knowledge and estimating the data volume required for effective SSL in SEM analysis. Overall, advancements are made in understanding SSL techniques in SEM image analysis, with practical tools and implementation guidelines provided. These contributions provide a comprehensive framework for effectively utilizing the large volumes of unlabeled SEM data generated by automated laboratories, exemplified by enhancing the efficiency and accuracy of particle segmentation.

## Results

### Dataset

**Fig. 2:**
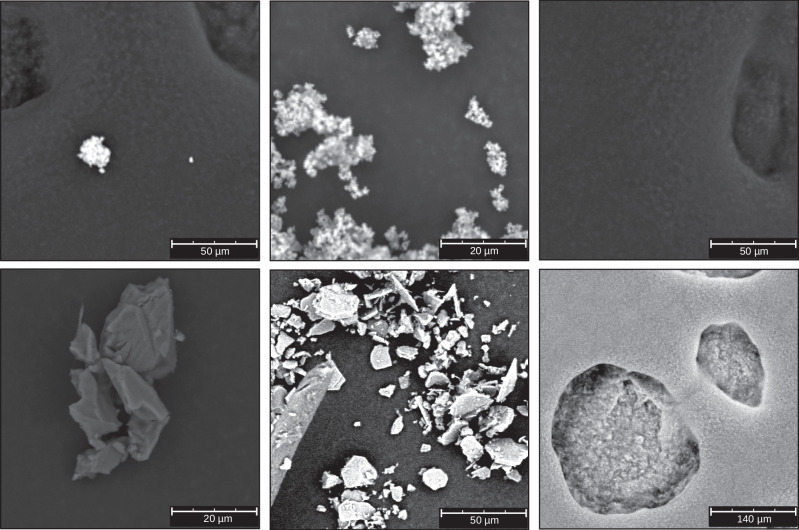
Six representative examples from the SSL dataset showcasing the diversity of samples. The dataset includes clear objects and structures (first two columns) and challenging samples with background noise, carbon tape features, or blurred elements (last column).

Two distinct datasets are utilized in this study to evaluate the effectiveness of SSL techniques in SEM image analysis: one for the SSL pretext training and another one for downstream application. For the SSL pretext training, a large-scale dataset consisting of 24,751 SEM images is generated. Figure 2 displays six examples, with clear objects and structures in the top row and challenging samples in the bottom row, showcasing the diversity of the dataset. This dataset was generated using a Thermofisher Phenom XL tabletop SEM, which captured single frames from entire samples in a rasterized fashion. Importantly, all captured images were retained without any filtering or selection process. As a result, the dataset encompasses a wide variety of image qualities, including well-defined particles, blurred specimens, and frames containing only background noise or carbon tape features. This deliberate inclusion of challenging and imperfect images makes the dataset a realistic and rigorous test case for SSL methods, mimicking the variability encountered in real-world SEM imaging scenarios. This extensive dataset remains unannotated, serving as a resource for unsupervised or self-supervised pretext tasks. For the downstream task of particle segmentation, a dataset comprising 91 SEM images featuring 10 different compounds is employed, which intentionally includes typical SEM challenges such as blur, particle overlap, and variable morphology, rather than idealized, high-quality images^3^. The deliberate inclusion of such challenging images enhances the practical relevance and robustness of potential results. Comprehensive details regarding the downstream dataset, including sample quality, composition, and annotation protocols, are provided in the publication where it was introduced initially^3^. These images were captured at both low (>50 *μ*m field of view, 51 images) and high (<50 *μ*m field of view, 40 images) magnifications, providing a range of particle sizes and morphologies. Expert annotators labeled the images for particle segmentation to ensure high-quality ground truth data. The dataset is divided into training, validation, and testing sets for robust model evaluation. There are 34 training, 8 validation, and 9 testing samples for low magnification. High magnification includes 25 training, 7 validation, and 8 testing samples.

### Experiment setup

To estimate how the different SSL paradigms compete, the performance of DenseCL^32^, which is established as one of the most effective CL approaches for segmentation tasks^22^, is compared against ConvNeXtV2 and ImageNet fine-tuning. For ensuring a fair comparison, the ConvNeXt^27^ architecture is used as encoders for all approaches. ConvNeXt, available in various scales from the Atto model with 3.4 million parameters to the Huge model with 657 million learnable parameters, is evaluated across its different variations to determine the optimal model size for SSL with SEM data. A comprehensive list of all backbone model sizes is available in Supplementary Table 1, and details regarding the ConvNeXt backbone and ConvNeXtV2 method are detailed in “ConvNeXtV2 architecture”.

The pretraining process is conducted differently for each method: *DenseCL* and *ConvNeXtV2* are pretrained on the unannotated dataset introduced in this study, consisting of 24,751 SEM images. For the *ImageNet* method, the pretraining is done through supervised classification of labeled ImageNet images. Further, a *No Pretraining* baseline model is included, in which no pretraining is used, and model parameters are initialized randomly before being trained from scratch on the labeled data. Following the pretraining phase, the encoders of the three methods (DenseCL, ConvNeXtV2, and ImageNet) and the random parameters (No Pretraining) are employed in a supervised downstream task using the separate labeled dataset^3^. The models are trained using the training and validation splits, while the final evaluation is conducted on the unseen test split to assess generalization performance.

To quantitatively compare the performance of the different approaches, the Aggregated Jaccard Index (AJI^+^)^33^ is selected as the quality metric. The AJI^+^ is chosen for its ability to comprehensively assess both localization accuracy and segmentation quality in instance segmentation tasks, making it particularly suitable for evaluating particle segmentation in SEM images. Commonly used metrics such as precision, recall, F1-score, or mAP50 are not applied here, as they evaluate detection or classification performance independently and may overlook errors arising from inaccurate instance boundaries. The AJI^+^, in contrast, jointly accounts for detection and segmentation quality, providing a single, interpretable score well aligned with the goals of this study.

Further, to assess the comparative effectiveness of the methods, the Error Reduction Rate (ERR)^34^ is used. This metric quantifies the relative decrease in error rates between two approaches, providing a clear measure of performance improvement. Details about the ERR are provided in “Methods”. Class Activation Maps (CAMs) and feature maps are utilized to evaluate the learning capabilities of the different approaches. Feature maps are particularly useful for particle segmentation in SEM images as they visualize the filters learned by the CNN, representing the activation of specific features detected by the network’s convolutional layers. This allows for a deeper understanding of how the model interprets particle characteristics. CAMs^35^ are employed to identify which parts of an SEM scan the respective CNN focuses on the most, providing insights into the model’s attention mechanisms. More details about feature maps are provided in “Methods”. Lastly, the instance particle detection quality is observed for individual objects by examining the actual segmentations generated by the approaches. This observation contributes to the overall evaluation by providing a direct assessment of the model’s ability to accurately delineate and identify individual particles, which is crucial in SEM image analysis. Through these combined methods, an all-encompassing view of the models is gained, allowing for a comprehensive evaluation of their performance in particle segmentation tasks.

### Comparative performance evaluation

**Fig. 3:**
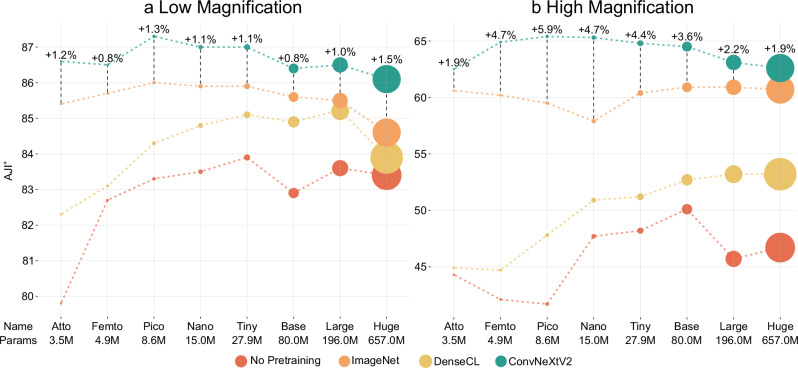
Comparison of No Pretraining, ImageNet, DenseCL, and ConvNeXtV2 with different backbone scales. Each circle represents one method’s AJI^+^ score in percentages on the supervised downstream task^3^. The size of the circles aligns with the number of parameters for the backbones. The difference between the best and second-best methods are provided at the circles of the ConvNeXtV2 model. The vertical axis is the AJI^+^ scores, and the horizontal axis is the respective backbone with the number of parameters in millions. See Supplementary Table 2 for the complete tabulated results.

The comparative performance evaluation illustrated in Fig. 3 provides insights into the impact of pretraining and model complexity on segmentation quality. The x-axis shows the backbone scales from Atto to Huge, and the y-axis displays the AJI^+^ scores in percentages for No Pretraining, ImageNet, DenseCL, and ConvNeXtV2. The AJI^+^s show an evident improvement with pretraining across all methods compared to the No Pretraining baseline. ConvNeXtV2 emerges as the clear leader, consistently surpassing both ImageNet pretraining and DenseCL across all backbone sizes for low and high magnification tasks. The performance of ConvNeXtV2 is particularly noticeable for small backbones. This gap between the methods narrows slightly for larger backbones but remains notable. Regarding the performance, the methods are arranged in the same way for all configurations: first ConvNeXtV2, followed by ImageNet, then DenseCL, and last, No Pretraining.

In low magnifications, shown in Fig. 3a, ImageNet demonstrates strong performance, situating itself as the second-best method after ConvNeXtV2. The gap between ConvNeXtV2 and ImageNet varies between +0.8% and +1.5%, which is remarkable for the range well above the 85% mark for the AJI^+^ score. DenseCL is distinctly below ImageNet and ConvNeXtV2 for smaller models but still noticeably better than No Pretraining. The gap becomes narrower with larger models, suggesting that for the low magnification task, the MAE approach of ConvNeXtV2 is more effective at learning useful representations than the CL approach of DenseCL as it needs more samples to come closer. The performance of ConvNeXtV2 peaks with the Pico backbone (87.3% AJI^+^) and remains relatively stable for larger models. Similar trends are also observable for the other methods, which indicates that for this particular task, increasing model size beyond Pico does not yield substantial benefits and may even degrade performance for very large models. Observing the relative improvement of the methods for the Pico backbone, a 24% ERR is calculated between No Pretraining and ConvNeXtV2, which means that errors are reduced by 24% relative to the baseline when ConvNeXtV2 is employed. Furthermore, compared to DenseCL, a 19% relative error reduction is demonstrated by ConvNeXtV2, while an 11% reduction is shown relative to ImageNet pretraining.

For high-magnification images (shown in Fig. 3b), the gaps between the methods become even more pronounced. The difference between ConvNeXtV2 and ImageNet is between +1.9% and +5.9%. Notably, DenseCL and No Pretraining perform significantly worse than ConvNeXtV2 and ImageNet in this high-magnification scenario. This stark performance gap suggests that the high-magnification dataset may not contain sufficient information for DenseCL to learn the relevant features effectively. In contrast, ConvNeXtV2 and ImageNet, seem to contain features that are transferable and beneficial for downstream training. This is especially striking as ConvNeXtV2 and DenseCL are trained on the same images, showing that ConvNeXtV2 extracts information from the data much more efficiently. Further, this proves that ConvNeXtV2’s MAE approach excels at capturing meaningful features for more complex segmentation tasks, as high magnification images in SEMs are often fuzzy and unclear. Again, as in low magnifications, medium-sized models (Pico backbone with 8.6M parameters) achieve noticeably better results. In contrast, larger models show diminishing returns or even decreased performance, emphasizing the importance of selecting the correct pretraining method and an appropriate number of trainable parameters and that more parameters may not result in better performance. The relative improvement with the ERR is even more striking in high magnifications. For the Pico backbone, the ERR between No Pretraining and ConvNeXtV2 is 41%, 34% compared to DenseCL and 15% with ImageNet.

These qualitative results demonstrate that pretraining substantially enhances performance quality, with the ConvNeXtV2 architecture proving especially effective for demanding SEM image analysis tasks. The performance of ConvNeXtV2 is particularly remarkable, as it surpasses even the strong baseline of ImageNet pretraining, which is especially striking considering that ConvNeXtV2’s training was conducted with about 25,000 samples, while ImageNet comprises more than 14 million data points. This achievement suggests that ConvNeXtV2 is learning highly relevant features during its pretraining phase. The results also emphasize that model size optimization, rather than maximization, is crucial for optimal performance in this context, as the smaller models provide the best results.

**Fig. 4:**
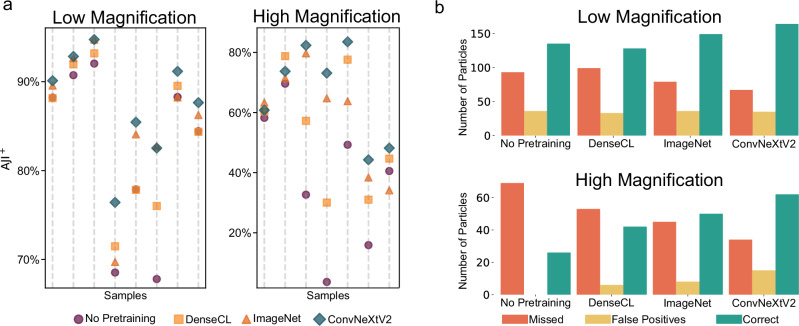
Details regarding all individual test samples for the Pico backbone. No Pretraining is training with random initialization of model weights, ImageNet is fine-tuning after training on the ImageNet classification challenge, and DenseCL / ConvNextV2 are the SSL methods pretrained on the dataset of this work. **a** The AJI^+^ scores in percentages for the individual samples of the test set for the downstream task. **b** The distribution of segmented particles for each method over all test set samples. A particle is correct if the Intersection over Union (IoU) between one ground truth and an estimated particle is above 40%. A ground-truth particle is considered missed if there is no estimated particle with an IoU ≥ 40% to it. An estimated particle is considered a false positive if there is no ground-truth particle with a IoU ≥ 40%.

The performance of the different approaches in more detail for the best ConvNeXtV2 backbone Pico is shown in Fig. 4. Figure 4a displays the AJI^+^ scores for all individual test samples and illustrates ConvNeXtV2’s consistent superiority across both low and high magnification samples. ConvNeXtV2 outperforms all other approaches at low magnification while also leading in most high magnification samples. Notably, in low magnification scenarios, the performance gap between methods narrows for samples that are easier to segment, whereas it widens significantly for more challenging samples. This disparity is particularly evident with ConvNeXtV2, demonstrating a clear advantage in handling complex cases. The importance of pretraining is further underscored by the notably poor performance of the No Pretraining approach, especially when confronted with complex samples. While the results for high magnification samples show more variability, ConvNeXtV2 still maintains a strong lead. Once again, results from the challenging samples highlight the benefits of pretraining, reinforcing its crucial role in enhancing segmentation performance across varying levels of sample complexity.

The distribution of correctly and incorrectly segmented particles is illustrated in Fig. 4b. ConvNeXtV2 exhibits the highest accuracy in particle identification for both magnifications, with a particularly pronounced advantage in high magnification scenarios. It also misses the least number of particles across both magnification levels. Interestingly, while the number of false positive particles is comparable among all methods for low-magnification images, ConvNeXtV2 shows a slightly higher rate of false positives in high magnification. This observation suggests an increased sensitivity of the ConvNeXtV2 method, which may be advantageous for detecting subtle features in complex, high-resolution SEM images but also makes the model more sensitive. Again, the impact of pretraining is evident, with ConvNeXtV2 demonstrating the most significant enhancement in ML model performance. In stark contrast, the No Pretraining approach consistently yields the poorest results, correctly identifying substantially fewer particles and missing a more significant number than ConvNeXtV2 and ImageNet pretraining methods. At low magnification, all methods achieve higher accuracy in identification than at high magnification, aligning with the understanding that high magnification SEM scans are more challenging, as evidenced by fewer correct identifications and more missed particles overall. These findings underscore the critical role of pretraining, particularly using advanced SSL techniques like ConvNeXtV2, in improving the accuracy and robustness of particle segmentation in SEM image analysis.

### Qualitative evaluation

**Fig. 5:**
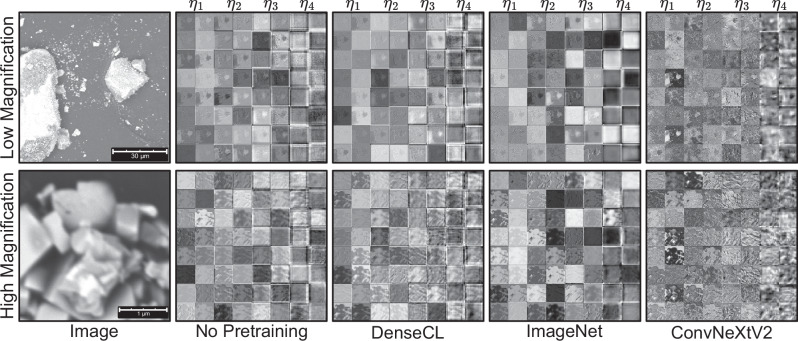
Visualization of feature activations from the Pico backbone. For each of the four ConvNeXt backbone stages *η*_*i*_ ∈ 1, …4, 16 randomly selected feature activation maps are shown in two columns in small squares (see “ConvNeXtV2 architecture” for details about the stages). The activation maps are drawn after the GRN layer, and the network weights are not modified after pretraining (architectural details are given in refs. ^27,28^).

Following the quantitative performance evaluation discussed in the previous section, the different approaches to segmentation are also compared visually. Figure 5 presents feature activation maps for two exemplary SEM images at low and high magnifications, comparing the different pretraining methods and random initialization (No Pretraining). These maps, drawn from the dimension-expansion layers following the Global Response Normalization block in the ConvNeXt backbone (Pico), offer insights into the network’s learned features and decision-making process. This network stage was selected since the feature collapse phenomenon can mainly be observed there^28^, which suggests that those features are particularly significant. Observing the learned features is important if neural networks are to be compared, as they offer insights into the quality and diversity of the learned knowledge. In this study, the visualization displays 16 randomly chosen features across the four hierarchical stages of the network (*η*_1_ to *η*_4_) with unmodified network weights after pretraining. Details about CNN stages and where they are located within the network are detailed in “ConvNeXtV2 architecture”. This visualization allows for a comparative analysis of the feature learning capabilities of different pretraining approaches. The network parameters of the pretraining methods are drawn from after the SSL pretext task before any supervised training. No Pretraining is random initialization of the network.

The learned features across the various pretraining methods clearly differ. The analysis shows a clear progression from DenseCL over ImageNet to ConvNeXtV2. DenseCL’s activation maps seem unstructured and repetitive, particularly in the early layers, indicating challenges in learning diverse and meaningful features from the SEM data. For the low magnification sample, the activations of DenseCL lack organization and often repeat, especially in *η*_1_ and *η*_2_. The activations display broad, low-contrast patterns that lack sharp edges. The diffuse characteristics of the early layer activations extend to the later layers (*η*_3_ and *η*_4_). Consequently, less defined features emerge throughout the network, and a clear, hierarchical progression of feature complexity from earlier to later layers is not evident. Importantly, the feature maps of DenseCL are evidently similar to random initialization (No Pretraining), further indicating an ineffective learning process. In high magnification images, DenseCL’s activations continue to be unfocused, with few recognizable patterns. This consistent lack of structure across magnifications points to a fundamental limitation in DenseCL’s ability to adjust to SEM data.

ImageNet pretraining demonstrates an improvement over DenseCL, featuring more defined patterns, although it remains less advanced compared to ConvNeXtV2. In low magnification samples, ImageNet reveals more distinct patterns than DenseCL, highlighting noticeable edge detection early on and more intricate features later. The overall feature maps differ significantly from those without pretraining. For low magnification, in the first layer (*η*_1_), ImageNet displays patterns similar to ConvNeXtV2, indicating similarly learned low-level features. However, in *η*_4_, ImageNet’s activations appear washed out with dead features, identifiable by uniform, almost completely black or white images. In high magnification images, ImageNet displays enhanced feature detection compared to DenseCL but is less accurate than in low magnification, suggesting challenges in adapting to higher magnification. Even more saturated or dead feature maps are visible than in the low magnification scenario.

ConvNeXtV2 demonstrates superior feature learning capabilities at all stages for both low and high magnifications, showcasing refined and hierarchical structures in its activation maps. This advanced understanding of image features is evident in the progression from early to later stages. For low magnification samples, ConvNeXtV2 reveals distinct object boundaries and internal structures, maintaining the clearest patterns in *η*_4_ while the activations of other methods become less defined. In high magnification images, it retains well-defined and structured activations, exhibiting robustness in handling inherent fuzziness and ambiguity. ConvNeXtV2 sets itself apart with texture-sensitive activation patterns, particularly in higher stages (*η*_3_ and *η*_4_), showcasing intricate, interconnected geometric patterns that resemble the material’s structure. These patterns include hexagonal arrangements, circular motifs, and fine-grained texture details, contrasting with the block-like structures displayed by other methods. The remarkable performance of ConvNeXtV2 across various magnifications and network stages indicates a practical and generalizable approach to SSL in the context of SEM image analysis.

**Fig. 6:**
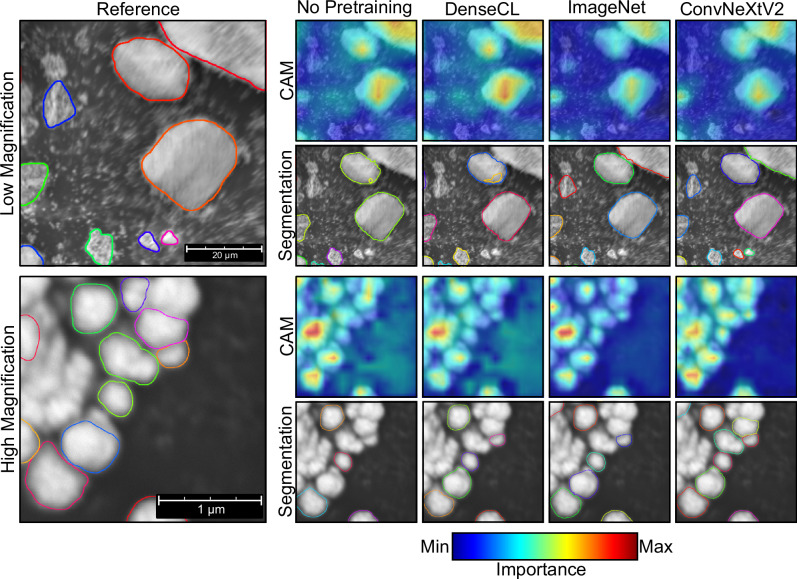
One sample image for both magnifications with ground truth labels (Reference). The colored curves are the domain experts' segmentations (left) or the respective methods (right). Each curve is color-coded to indicate the individual instances. Further, the Class Activation Maps (CAMs)^35^ display which parts of the image the methods focus on the most.

Figure 6 visually compares the segmentation performance and feature focus for the considered methods across both magnifications in the dataset. CAMs are utilized to visualize the regions of the input image most influential in the model’s decision-making process, providing insights into which parts of the image the neural network focuses on most strongly by highlighting areas of high importance in red and yellow and areas of low importance in blue. Further, ground-truth segmentations are provided as a reference for both magnifications, alongside the segmentation results for each method. Unlike in Fig. 5, where only the pretext task quality was observed, the networks are now evaluated after the supervised downstream task.

The CAMs reveal distinct focus patterns across the different methods. No Pretraining exhibits scattered activation patterns with multiple yellow-green regions that extend beyond particle boundaries, indicating a poor ability to localize important regions within the images. DenseCL shows a marginal improvement, with slightly more focused CAMs, but still lacks precision in highlighting key regions of interest. ImageNet pretraining demonstrates a notable enhancement, with CAMs showing an improved focus on central regions of interest. ConvNeXtV2 is the most concentrated and precise CAMs among all methods. ConvNeXtV2’s CAMs exhibit focused activation patterns shown by the most focused yellow-red regions that precisely align with particle boundaries while maintaining consistently low activations (deep blue) in background areas, suggesting a superior ability to identify and emphasize the most relevant image features for the segmentation task.

The difference between the methods is most visible in the high magnification case, where No Pretraining and DenseCL exhibit insufficient focus. ImageNet and especially ConvNeXtV2 concentrate on the region where particles are present. The segmentation quality closely mirrors the trends observed in the CAMs. No Pretraining yields the poorest segmentation results, missing numerous particles and failing to accurately delineate particle boundaries, with most smaller particles not being segmented, but also larger ones are ignored. Further, the contours of the correctly identified particles are irregular compared to the ground-truth reference. DenseCL shows a slight improvement but still produces inaccurate segmentations with mismatched boundaries. ImageNet shows a noticeable improvement compared to No Pretraining and DenseCL, demonstrating improved accuracy, less diffuse boundaries, and more plausible detected particles. ConvNeXtV2, consistent with its superior CAM performance, provides the highest segmentation quality among all methods. Its segmentation results closely match the ground truth contours, accurately capturing particle shapes and boundaries.

A detailed analysis of segmentation quality across the two example images further shows the differences between the pretraining methods. In the low magnification sample, No Pretraining and DenseCL produce similar masks, overlooking smaller particles at the bottom while erroneously identifying a non-existent particle within a larger one at the top. ImageNet displays improved overall segmentation but misses the two smallest particles at the bottom. ConvNeXtV2 outperforms the others, locating all particles, including the smallest ones. The high magnification case further emphasizes the differences, with No Pretraining, DenseCL, and ImageNet failing to identify numerous particles. ConvNeXtV2 maintains its superior performance, segmenting the most particles even in this challenging, clustered image. These visual results corroborate the quantitative findings, demonstrating ConvNeXtV2’s superior performance in feature localization and segmentation accuracy. Its consistent precision across magnifications highlights its robustness in handling SEM image complexities, especially in challenging high magnification scenarios with complex and ambiguous particle morphologies.

### Ablation study

**Fig. 7:**
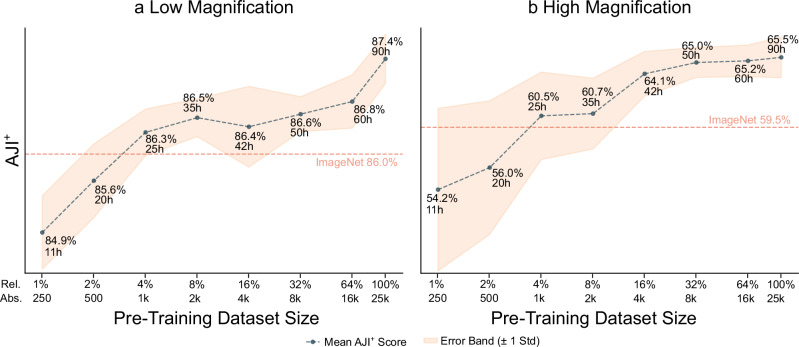
AJI^+^ compared to pretraining dataset size. The horizontal axis is the number of samples available for SSL training of ConvNeXtV2 with the Pico backbone with a log2 scale, and the vertical axis is the corresponding AJI^+^ score in percentages after downstream training. The standard error bands are plotted along the scores, and ImageNet fine-tuning AJI^+^ score is provided for comparison. The experiment is repeated 10 times. See Supplementary Table 3 for the complete tabulated results.

An ablation study is conducted using varying amounts of training data for the pretext task to optimize the ConvNeXtV2 pretraining method with SEM data and gain a deeper understanding of the impact of pretraining dataset size. Figure 7 illustrates the relationship between the number of samples used for SSL pretraining of ConvNeXtV2 Pico and the resulting AJI^+^ scores for both low and high magnifications after the supervised downstream training. The sizes of the datasets are determined by the formula: *ρ*_d_ = 2^*s*^/100, where *s* varies from 0 to 6. This results in dataset sizes ranging from 1% to 64% of the original dataset, focusing on smaller sizes to identify whether significantly more samples correlate with equally higher performance.

For low magnification tasks, the AJI^+^ improves consistently as the dataset size increases, with ConvNeXtV2 surpassing ImageNet fine-tuning performance using only 1000 samples (about 4% of the total dataset). This demonstrates the efficiency of ConvNeXtV2 in learning useful representations even with limited data. The performance continues to improve steadily, with a considerable jump between 16,000 samples (64% of the dataset) and the full dataset, suggesting that further increases in sample size may yield additional enhancements in segmentation quality. The standard deviation is consistently low, between 0.2 and 0.6 for all splits. For high magnification tasks, the impact of increased pretraining data is even more pronounced, with the AJI^+^ score demonstrating an increase of over 10% when comparing the performance from using just 1% of the data to that achieved with the full dataset. As with low magnification, a minimum of 1000 samples is required to exceed the baseline established by ImageNet fine-tuning. However, performance gains begin to saturate beyond 32% of the total dataset size, implying that further increases in data volume are unlikely to result in substantial enhancements in model performance. In the high magnification case, the standard deviation narrows for higher sample counts, meaning that a more consistent performance across different runs is achieved as the dataset size increases, indicating improved feature learning.

The distinct performance curves for low and high magnification tasks provide valuable insights for assessing dataset sizes. In high magnification tasks, the sharp increase from 500 to 1000 pretraining samples, followed by diminishing returns after 4000 samples, suggests an optimal dataset size of 4000 to 8000 samples for cost-effective training. This critical mass of data is essential for effectively learning high magnification features, as evidenced by the narrowing standard deviation band. The noticeable performance saturation beyond 64% of the total dataset size indicates that further data expansion may not result in significant improvements. In contrast, the low magnification curve’s gradual and consistent improvement, with no clear saturation point, suggests that larger datasets might continue to enhance performance. These observations underscore the importance of tailoring dataset sizes to specific magnification levels and task requirements. These results emphasize how balancing dataset size with computational resources and expected performance gains is vital, recognizing that optimal dataset sizes may vary across different length scales in SEM image analysis. Furthermore, if the data comprises lower magnification images, more pretraining samples may be required compared to high magnification ones.

The results from this ablation study provide critical insights into the optimal configuration for SSL in SEM image analysis. While larger datasets generally lead to better model performance, a threshold exists beyond which additional data yields diminishing returns. Especially for high magnification data, more than 25,000 data points do not seem to be necessary. This finding underscores the necessity for carefully considering dataset size during the pretraining phase to maximize efficiency and effectiveness in segmentation tasks.

## Discussion

The present study demonstrates the significant potential of SSL in SEM imaging by assessing various approaches in a structured manner, demonstrated on the challenging task of particle segmentation for powder samples.

The comparative performance evaluation reveals that all pretraining methods consistently outperform the No Pretraining baseline, underscoring the value of SSL in leveraging unlabeled SEM datasets. Among the evaluated methods, ConvNeXtV2 emerges as the superior approach, consistently achieving the highest AJI^+^ scores across all backbone sizes and magnification levels, outperforming both the state-of-the-art CL approach DenseCL and the popular ImageNet pretraining method, making it the most promising approach for applications within SEM analysis. Notably, ImageNet pretraining consistently outperforms DenseCL, which challenges the common assumption that domain-specific pretraining yields the best results, as DenseCL was trained on SEM data. This may be due to the great diversity and scale of the ImageNet dataset, which allows for the learning of more generalized and transferable visual features, even for highly specialized tasks such as SEM particle segmentation.

The superiority of ConvNeXtV2 is particularly noteworthy given that it was trained on a relatively small dataset of about 25,000 SEM images, in contrast to the 14 million images used in ImageNet pretraining. The performance gap between ConvNeXtV2 and other methods widens for high magnification tasks, suggesting that the MAE approach employed by ConvNeXtV2 is particularly effective at capturing relevant features in complex, high-resolution SEM images. Furthermore, the quantitative results demonstrate that the MAE approach for SSL achieves notably better results than the currently prevalent CL paradigm.

The effectiveness of ConvNeXtV2 is further corroborated by the qualitative analysis of feature activation maps and CAMs. ConvNeXtV2 demonstrates the most refined and hierarchical feature learning across all network stages, learning recognizable better features in the CNN than DenseCL and ImageNet. The CAMs show precise alignment with particle boundaries, verifying the superior ability to identify and emphasize the most relevant image features. This results in the most fitting segmentations of objects and detection of small or unclear particles where other methods fail, particularly in challenging high magnification scenarios with complex and ambiguous morphologies.

Interestingly, medium-sized models, such as the Pico backbone with 8.6 million parameters, often achieve the best results, while larger models show diminishing returns or decreased performance. This finding challenges the assumption that larger models invariably lead to better performance, especially in the context of SSL with specialized datasets. This suggests that for SEM image analysis tasks, there is an optimal balance between model complexity and performance, with simpler models often outperforming complex ones. This means that practitioners should begin with smaller architectures to effectively identify the point where more trainable parameters yield diminishing returns. High model complexity does not necessarily improve performance and could lead to degradation, as the models seem to reach a saturation point where additional parameters fail to capture new meaningful knowledge and instead begin fitting random noise in the training data that doesn’t represent true underlying distributions.

The ablation study on dataset size reveals important insights into the data requirements for effective SSL in SEM image analysis. For low magnification tasks, ConvNeXtV2 surpasses ImageNet fine-tuning performance using only 1000 samples, demonstrating its efficiency in learning useful representations even with limited data. The performance curve for low magnification tasks shows a gradual and consistent improvement, potentially indicating the learning of richer and more diverse features with increasing dataset size. The impact of increased pretraining data is even more pronounced for high magnification tasks, suggesting a critical mass of data between 4000 and 8000 samples is needed to effectively learn high magnification features. However, performance gains begin to saturate beyond 64% of the total dataset size, implying that further increases in data volume are unlikely to result in substantial enhancements in model performance for high magnification tasks.

The results of the presented study complement recent advances in lab automation, suggesting that SSL pipelines could be integrated with autonomous workflows for material synthesis and characterization while requiring minimal human effort. ConvNeXtV2’s ability to achieve superior performance with relatively small datasets suggests that it is particularly valuable in domains where large annotated datasets are difficult or expensive to obtain. Furthermore, the observed optimal model sizes and performance plateaus with varying data volumes guide optimal dataset sizes for SSL pretraining, potentially saving computational resources and time in future studies. While this study focuses on inorganic powder SEM images, the trained ConvNeXtV2 model establishes a strong baseline that is likely applicable to other material systems. Due to domain-specific variations in imaging modalities and data characteristics, additional fine-tuning may be needed to optimize performance in these new contexts. Nonetheless, our models provide a robust starting point, requiring minimal adjustments for broad applicability.

The superior performance of ConvNeXtV2 across various metrics and tasks suggests that the MAE approach is particularly well-suited for learning meaningful representations from SEM data. This could be attributed to the method’s ability to capture both local and global context in images, which is crucial for accurate particle segmentation in complex SEM images.

While the results are promising, it is essential to note some limitations of the present study. Firstly, the current speed of ML-based segmentation could be a limiting factor as segmenting a single image may take up to several seconds (Evaluating an image measuring 1920 × 1200 pixels takes approximately nine seconds to process using the ConvNeXtV2 Pico backbone within the Mask-RCNN model exported as an ONNX executable and running on a desktop SEM on a Intel(R) Core(TM) i5-10500 CPU @ 3.10GHz.); future work should optimize model efficiency, which is an important consideration for real-time analysis in automated laboratory settings.

Further, the evaluation was conducted on a specific set of SEM images, and further research is needed to confirm the generalizability of these findings across a broader range of materials and imaging conditions. Also, further investigating the theoretical implications of the MAE approach’s effectiveness in SSL for image analysis tasks could provide deeper insights into its success.

The findings of the present study offer valuable insights into the optimal configuration of SSL methods for materials characterization and present in-depth approaches for reliably evaluating these methods, paving the way for more efficient and accurate data analysis in materials science research and industrial applications.

## Methods

### ConvNeXtV2 architecture

**Fig. 8:**
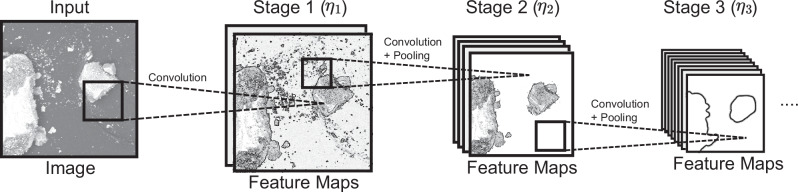
General CNN architecture. The input image undergoes multiple convolutions to generate initial feature maps in stage 1 (*η*_1_), which are then convoluted and pooled numerous times in the following stages to create hierarchical features.

Figure 8 illustrates how CNNs learn to detect objects through hierarchical feature learning. CNNs process images in stages, building increasingly complex representations. Initial convolutional layers detect basic features, while intermediate stages combine these to recognize complex patterns. Later stages capture high-level, task-specific features, aggregating fine details into broader concepts. This hierarchical approach allows CNNs to capture intricate details and global context (see ref. ^36^ for an in-depth explanation of CNNs). In classic CNNs, the layers are strictly sequential, and features from previous layers must always be processed via other learned features in deeper layers of the network. This structure can lead to vanishing gradients, degradation problems, and limited feature propagation, drastically limiting the performance and depth networks can achieve^37^. ResNets enhance CNNs by introducing residual blocks with shortcut connections, allowing deeper networks to be trained more effectively. These skip connections enable direct feature propagation, easier optimization, and improved gradient flow, addressing the limitations of classical CNNs and allowing for superior performance with much deeper architectures.

ConvNeXt^28^ builds on top of ResNets^37^ by addressing recent research that has shown that ViTs outperform CNNs if data is sufficient^38^. Hence, ConvNeXt modernizes ResNets by integrating insights from ViTs while retaining the efficiency of CNNs. It achieves performance improvements through high-level structural and low-level design changes, significantly narrowing the gap between CNNs and ViTs. The changes include redistributing computational blocks across network stages inspired by transformers^39^, introducing a stem block with 4 × 4 non-overlapping convolutions at the beginning of the network, layer normalization for efficient input downsampling, and adopting depthwise convolutions to reduce floating-point operations while increasing network capacity. Inspired by transformer blocks and MobileNetV2^40^, ConvNeXt employs an inverted bottleneck structure, expanding hidden dimensions to four times the input dimension, and uses larger kernel sizes (up to 7 × 7) to increase the receptive field. Further, the Rectified Linear Unit (ReLU) activation is replaced with Gaussian Error Linear Unit (GELU) for better performance, using layer normalization instead of batch normalization for alignment with transformer architectures and introducing separate downsampling layers with 2 × 2 convolutions and normalization to preserve spatial information during downsampling. These enhancements enable ConvNeXt to rival or surpass transformer-based models while maintaining the simplicity and efficiency of CNNs. ConvNeXt is available in scales ranging from 3.7 million to 198 million parameters, serving diverse computational needs.

ConvNeXtV2^28^ further develops ConvNeXt by enabling SSL pretraining, primarily through two components: Fully Convolutional Masked Autoencoder (FCMAE) training and Global Response Normalization (GRN).. The FCMAE enables MAEs for CNNs using the ConvNeXt architecture, overcoming prior limitations in SSL^41^. It processes masked input patches from the stem block using sparse convolutions, preventing mask pattern dissipation during hierarchical convolution and forcing the network to generalize image structures by extracting features from partially obscured images and reconstructing the original image via a lightweight decoder. This reconstruction task helps the network learn meaningful features even with missing data. The GRN enhances inter-channel feature competition and mitigates feature collapse during training on masked inputs. It computes global statistics across spatial dimensions and selected channels, normalizes feature responses, and applies a learnable affine transformation. Integrated into ConvNeXtV2 blocks between two convolutions, the GRN promotes diverse feature representations essential for supervised and self-supervised tasks. Together, the FCMAE enables effective learning from unannotated data via MAE-based SSL, while the GRN ensures robust feature diversity. Like its predecessor, ConvNeXtV2 is available in multiple scales.

### DenseCL architecture

Dense Contrastive Learning (DenseCL) is a self-supervised learning framework designed explicitly for dense prediction tasks, such as particle segmentation^32^. Unlike traditional contrastive learning methods focusing on global image representations, DenseCL operates at the pixel or local feature level. It introduces a dense projection head that outputs dense feature vectors preserving spatial information, rather than a single global vector. During training, DenseCL optimizes a pairwise contrastive loss between corresponding local features across two augmented views of the same image. Positive pairs are identified by matching features with high cosine similarity, while negative pairs consist of local features from different images. This approach enables the model to learn fine-grained, spatially-aware features essential for dense tasks. The backbone and projection heads are trained end-to-end to maximize the similarity of corresponding local features and the dissimilarity of unrelated ones. DenseCL has been demonstrated to improve performance on dense prediction tasks, including object detection and segmentation, with minimal computational overhead compared to global contrastive methods^22^. This makes DenseCL particularly suitable for self-supervised pretraining in SEM image analysis, where detailed spatial understanding is crucial.

### Training details

All architectures and experiments are implemented in Python using PyTorch Lightning and Torchvision. The Minkowski Engine^42^ is used for sparse convolutions. For CAMs, the PyTorch Grad-CAM^43^ library is used. We conduct training on the supercomputer system Hochleistungsrechner Karlsruhe (HoreKa) at KIT. One computational node of HoreKa is equipped with an Intel Xeon Platinum 8368 CPU (2 sockets, 76 cores per socket) and four NVIDIA A100 Tensor Core GPUs. The SSL pretraining was conducted with distributed training using 20 nodes, each with 4 GPUs, resulting in a total of 80 GPUs, utilizing SLURM for job scheduling and PyTorch’s distributed training capabilities. For pretraining, the novel dataset (see Section 2.1) is split into an 80% training and 20% evaluation. A test split is not employed during pretraining. For DenseCL pretraining, we use the configuration suggested by the authors of the method, with a learning rate of 0.3, linear scaling with batch size, step-wise decay scheduling, and linear warm-up, along with SGD optimization and mixed-precision training^32^. The data augmentation techniques employed for DenseCL are the optimal ones found in ref. ^22^. Similarly, for ConvNeXtV2, we follow the creators’ suggestions, using a cosine decay learning rate schedule with a base rate of 8e-4, an AdamW optimizer with weight decay, and layer-wise learning rate decay^28^. Augmentations are adjusted to ensure fine-grained details and small particles are still visible. Hence, the images are not scaled but randomly cropped to 640 × 640 pixels and horizontally flipped with a chance of 50%. The patch size for the MAE masking is 32 × 32 pixels. In a previously performed hyperparameter study, the number of removed patches was optimized to 50%. Both SSL methods are trained for 15.000 epochs without early stopping. Expanding on the per-epoch durations listed in the Supplementary Table 1, total training times range from roughly 33 hours for the Atto model to about 188 hours for the Huge model.

After training, the epoch with the lowest validation loss is used for downstream training. The ImageNet weights are downloaded from the official ConvNeXtV2 GitHub page. Downstream training is conducted the same way for all methods. The Mask R-CNN implementation and configuration of^3^ is used, but the uncertainty head is not employed. The downstream experiments were repeated 10 times to obtain reliable metrics.

### Error Reduction Rate

The Error Reduction Rate (ERR)^34^ is utilized to evaluate the relative difference in error rates between different systems or methods. This metric is employed to quantify the extent to which the error rate has been diminished for a particular method *ϵ*_m_ when compared to a baseline *ϵ*_b_. The calculation of the ERR is performed as

1 $$\text{ERR} =\frac{{\epsilon}_{\rm{b}}-{\epsilon}_{\rm{m}}}{{\epsilon}_{\rm{b}}}* 100.$$

The outcome of this calculation is expressed as a percentage, representing the proportional decrease in errors achieved.

## Supplementary information

Supplementary Information

## Data Availability

The code for the adjusted ConvNeXtV2 model can be found at https://github.com/lrettenberger/ConvNeXt-V2. The Mask R-CNN model used is located at https://github.com/lrettenberger/Uncertainty-Aware-Particle-Segmentation-for-SEM. The novel dataset and ConvNeXtV2 pretraining weights can be downloaded at https://osf.io/yzrfv/.
