# Supplementary material for: Leveraging unlabeled SEM datasets with self-supervised learning for enhanced particle segmentation
Source: NPJ Comput Mater. 2025 Sep 22;11(1):289. doi: 10.1038/s41524-025-01802-3 (PMC12454128; doi:10.1038/s41524-025-01802-3)
Supplement: Supplementary file 1 — Supplementary Information [file 41524_2025_1802_MOESM1_ESM.pdf]

Supplementary Note 1: ConvNeXt model sizes

Supplementary Table 1 shows the backbone and ConvNeXtV2 model sizes, how the backbone accounts for the most significant portion of the ConvNeXtV2 model, and training times for one epoch on the HoreKa supercomputer.

		**#Parameters**		
**Name**	**Short Name**	**Backbone**	**ConvNeXtV2**	**Time / Epoch**
ConvNeXt Atto	Atto	3.40×10^6	3.70×10^6	8 Seconds
ConvNeXt Femto	Femto	4.90×10^6	5.20×10^6	9 Seconds
ConvNeXt Pico	Pico	8.60×10^6	9.10×10^6	11 Seconds
ConvNeXt Nano	Nano	1.50×10^7	1.60×10^7	13 Seconds
ConvNeXt Tiny	Tiny	2.79×10^7	2.86×10^7	14 Seconds
ConvNeXt Base	Base	8.80×10^7	8.90×10^7	18 Seconds
ConvNeXt Large	Large	1.96×10^8	1.98×10^8	26 Seconds
ConvNeXt Huge	Huge	6.57×10^8	6.60×10^8	45 Seconds

Supplementary Table 1 All ConvNeXt backbones and ConvNeXtV2 model sizes. *Name* is the full name of the respective backbone, *Short Name* the abbreviation used in this work, *#Parameters* the number of parameters for the *backbone* and the whole ConvNeXtV2 model respective, and *Time / Epoch* the time needed for training one epoch on the HoreKa supercomputer using 20 nodes (80 NVIDIA A100 Tensor Code GPUs).

Supplementary Note 2: Tabulated results comparative performance evaluation

Supplementary Table 2 lists the full tabulated results of the comparative performance evaluation in Section 2.3.

a) Low Magnification

Backbone	No Pretraining	DenseCL	ImageNet	ConvNeXtV2
Atto	79.8 ± 3.4	82.3 ± 2.2	85.4 ± 0.7	86.6 ± 0.3
Femto	82.7 ± 1.1	83.1 ± 0.9	85.7 ± 0.9	86.5 ± 0.6
Pico	83.3 ± 1.2	84.3 ± 0.7	86.0 ± 0.8	87.3 ± 0.4
Nano	83.5 ± 0.8	84.8 ± 0.7	85.9 ± 0.7	87.0 ± 0.4
Tiny	83.9 ± 0.6	85.1 ± 0.5	85.9 ± 0.8	87.0 ± 0.4
Base	82.9 ± 1.1	84.9 ± 0.7	85.6 ± 0.9	86.4 ± 1.3
Large	83.6 ± 0.7	85.2 ± 1.0	85.5 ± 0.5	86.5 ± 0.5
Huge	83.4 ± 0.7	83.9 ± 0.9	84.6 ± 1.0	86.1 ± 0.5

b) High Magnification

Backbone	No Pretraining	DenseCL	ImageNet	ConvNeXtV2
Atto	44.3 ± 5.8	44.9 ± 5.6	60.6 ± 2.1	62.5 ± 4.5
Femto	42.1 ± 5.8	44.7 ± 6.5	60.2 ± 3.7	64.9 ± 2.1
Pico	41.7 ± 9.1	47.8 ± 7.3	59.5 ± 3.5	65.4 ± 1.7
Nano	47.7 ± 5.4	50.9 ± 3.2	57.9 ± 3.1	65.3 ± 1.3
Tiny	48.2 ± 7.9	51.2 ± 4.8	60.4 ± 2.4	64.8 ± 2.7
Base	50.1 ± 5.7	52.7 ± 5.0	60.9 ± 2.8	64.5 ± 2.2
Large	45.7 ± 9.2	53.2 ± 6.2	60.9 ± 6.2	63.1 ± 2.4
Huge	46.7 ± 9.8	53.2 ± 4.3	60.7 ± 5.7	62.6 ± 2.0

Supplementary Table 2 Comparison of random initialization of the ConvNeXt backbone (*No Pretraining*), and pretraining with *DenseCL*, *ImageNet*, and the *ConvNeXtV2* approach. The methods are evaluated with the AJI^+ metric and all scales of the ConvNeXt backbone for the high- and low-magnification dataset splits of the downstream dataset. *DenseCL* and *ConvNeXtV2* are pre-trained with the novel dataset of this work, *ImageNet* is transfer learning from the *ImageNet* classification challenge, and *No Pretraining* is the baseline with randomly initialized weights. All configurations are repeated 10 times with random seeds, and the standard deviation is listed along the AJI^+.

Supplementary Note 3: Tabulated results ablation study

Supplementary Table 3 lists the full tabulated results of the ablation study in Section 2.5.

Magnification	Dataset Size (Relative)							
	**1%**	**2%**	**4%**	**8%**	**16%**	**32%**	**64%**	**100%**
**Low**	84.9 ± 0.5	85.6 ± 0.5	86.3 ± 0.3	86.5 ± 0.3	86.4 ± 0.6	86.6 ± 0.2	86.8 ± 0.4	87.4 ± 0.4
**High**	54.2 ± 6.9	56.0 ± 5.7	60.5 ± 3.7	60.7 ± 3.0	64.1 ± 1.9	65.0 ± 1.3	65.2 ± 1.4	65.5 ± 1.7

Supplementary Table 3 Comparison of different pertaining dataset sizes for the ConvNeXt Pico backbone. All experiments are repeated 10 times with random seeds, and the standard deviation is listed along the AJI^+.
